# A structured narrative review on VNS-treated drug-resistant epilepsy: EEG markers, neurochemical mechanisms, and future biomarker-driven computational directions

**DOI:** 10.3389/fnins.2026.1813014

**Published:** 2026-04-28

**Authors:** Antonella Muroni, Krishna Moorthi Bhat, Alberto Maleci, Francesco Marrosu

**Affiliations:** 1 Neurology Unit, Azienda Ospedaliero-Universitaria of Cagliari, Cagliari, Italy; 2 Department of Biomedical Engineering, Heersink School of Medicine and School of Engineering, University of Alabama at Birmingham, Birmingham, AL, United States; 3 Department of Medical Sciences and Public Health, University of Cagliari, Cagliari, Italy

**Keywords:** electroencephalography (EEG), epilepsy, neurochemical, neuromodulators, neurotransmitters, vagus nerve stimulation (VNS)

## Abstract

Vagus nerve stimulation (VNS) is an established adjunctive therapy for drug-resistant epilepsy. Experimental and clinical evidence indicates that its therapeutic effects involve distributed brain networks and multiple neurochemical pathways. Electroencephalography (EEG) has been widely used to characterize VNS-related neurophysiological changes, including alterations in conventional oscillatory activity, functional connectivity, and, more recently, aperiodic spectral components such as the spectral exponent and spectral offset. However, these EEG findings are often interpreted without sufficient consideration of the neurochemical intermediates that may contribute to the observed electrophysiological changes. In this structured narrative review, primarily focused on epilepsy, we examine how VNS-related EEG findings can be interpreted in light of noradrenergic, serotonergic, cholinergic, GABAergic, and neurotrophic mechanisms. We also discuss methodological challenges in the analysis of periodic and aperiodic EEG components and outline how machine learning approaches and adaptive closed-loop neuromodulation strategies may support the development of clinically useful VNS biomarkers.

## 1 Introduction

### 1.1 Clinical role of VNS and EEG in pharmacoresistant epilepsy

Vagus nerve stimulation (VNS) is a well-established therapeutic option for pharmacoresistant epilepsy (Ben-Menachem et al., 1994; George et al., 2000). Several studies have shown that, alongside seizure reduction, VNS can decrease interictal epileptiform activity (Kuba et al., 2002), reshape band-limited EEG rhythms by suppressing theta activity and enhancing gamma oscillations (Marrosu et al., 2005), and reorganize large-scale functional brain connectivity (Fraschini et al., 2014). More recently, a complementary line of research has focused on the characterization of the aperiodic, or scale-free (1/f-like), component of the EEG power spectrum (Donoghue, 2025; Donoghue et al., 2020). This broadband background activity, typically parameterized by the spectral exponent (slope) and spectral offset, has been proposed to reflect population-level excitation–inhibition balance, neuronal firing rate, and arousal states. These factors are particularly relevant for understanding global network effects associated with seizures and their treatment (Gerster et al., 2022; Charlebois et al., 2024; Salvatore et al., 2024). Despite these advances, EEG findings are often interpreted using simplified outcome categories, such as responder versus non-responder status, when comparing pre- and post-implant recordings (Coa et al., 2022; Yang et al., 2024). Such a binary framework may obscure the biological mechanisms underlying the observed electrophysiological changes. In particular, it raises the possibility that intermediate processes, including neurochemical modulation triggered or enhanced by VNS, may provide deeper insight into alterations in cortical electrogenesis. Because both epilepsy and neuromodulation fundamentally involve the regulation of cortical excitability, integrating periodic and aperiodic EEG metrics with biochemical mechanisms is both timely and of practical relevance. In routine clinical practice, VNS therapy is typically combined with first- and second-line anti-seizure medications (ASMs) (Perucca et al., 2024), particularly in drug-refractory cases. Since these therapeutic combinations require ongoing optimization (Arcand et al., 2017), a clearer understanding of both the biochemical and electrophysiological effects of VNS may contribute to improved ASM management. Although VNS has gained broader clinical interest beyond epilepsy, including applications in depression (Howland, 2014; Austelle et al., 2022), Alzheimer’s disease (Vargas-Caballero et al., 2022), cerebellar tremor (Marrosu et al., 2007), migraine (Hord et al., 2003), schizophrenia (Smucny et al., 2015), heroin-induced place preference and reduction of body weight and fat mass in experimental models (Bai et al., 2025; Banni et al., 2012), the present review focuses primarily on drug-resistant epilepsy. This focus reflects the fact that EEG biomarker research is currently most advanced in epilepsy and that implanted VNS therapy is most firmly established in this context. This perspective is also consistent with recent reviews addressing predictors of VNS response in epilepsy. Within this framework, alterations in frequency spectra and large-scale network activity become particularly informative when interpreted in relation to underlying neurochemical modulation.

### 1.2 Structured narrative approach

This narrative review was conducted using a structured search of the biomedical literature to identify studies investigating the electrophysiological and neurochemical effects of vagus nerve stimulation (VNS), with particular attention to electroencephalographic (EEG) biomarkers. Electronic databases, including PubMed, Scopus, and Web of Science, were searched for publications between 1990 and 2025 (with inclusion of selected earlier foundational studies), using combinations of keywords such as *vagus nerve stimulation*, *EEG*, *epilepsy*, *neuromodulation*, *functional connectivity*, *spectral analysis*, *aperiodic activity*, *neurotransmitters*, and *machine learning*.

**Figure 1:**
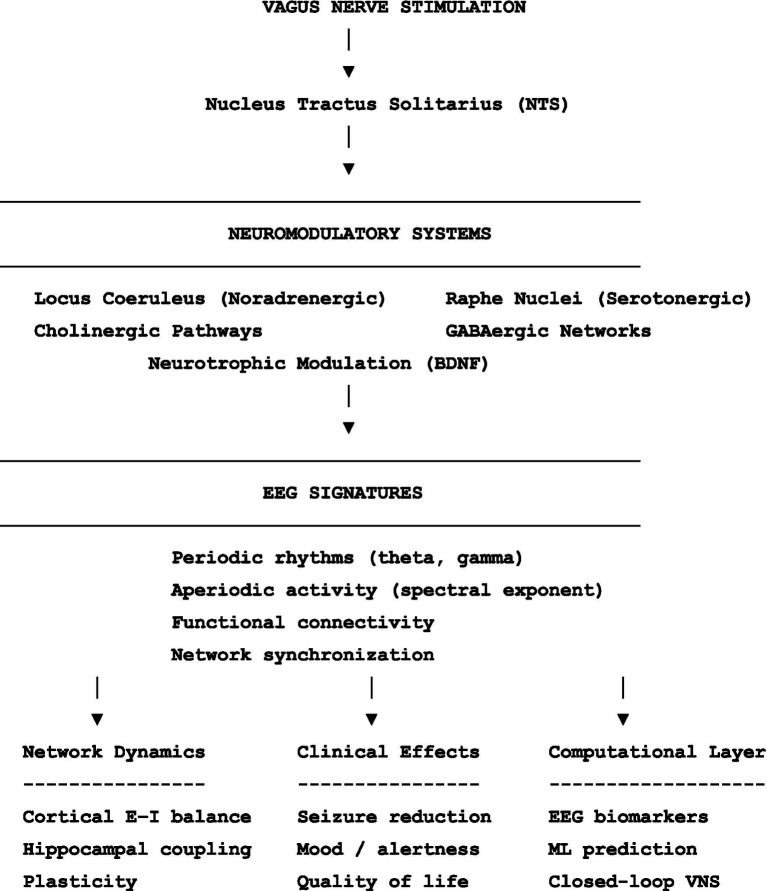
Flow chart of the multilevel framework linking vagus nerve stimulation, neuromodulatory mechanisms, EEG signatures, and translational applications. Vagus nerve stimulation activates afferent projections to the nucleus of the solitary tract, which in turn engages multiple neuromodulatory systems including noradrenergic, serotonergic, cholinergic, and GABAergic pathways as well as neurotrophic mechanisms such as BDNF. These pathways influence cortical excitability, synchronization, and network plasticity. Such effects may be reflected in electrophysiological signatures measurable with EEG, including periodic oscillations, aperiodic spectral components, and functional connectivity patterns. These signals provide a bridge between underlying neurochemical mechanisms and clinical outcomes, and may support the development of computational approaches for response prediction and adaptive neuromodulation strategies.

Studies were included if they examined: EEG changes associated with VNS, neurochemical mechanisms of VNS, computational approaches for analyzing neuromodulation-related EEG signals. Both clinical studies in humans and mechanistic studies in animal models were considered when relevant to understanding VNS-induced neuromodulatory processes. Articles focusing exclusively on unrelated neuromodulation techniques or lacking electrophysiological relevance were excluded. The selected literature was evaluated to synthesize current evidence linking VNS-induced neuromodulatory pathways, neurochemical dynamics, and EEG signatures, as well as emerging computational approaches aimed at identifying predictive biomarkers for neuromodulation therapies (Figure 1).

## 2 Experimental studies in historical perspective

### 2.1 Early attempts, abandoned concepts, and experimental neurophysiology

Historical accounts of vagus nerve stimulation (Lanska, 2002) indicate that Lanska (2002) was among the first to apply a rudimentary external device aimed at stimulating the vagus nerve to reduce what was then described as “venous hyperaemia,” considered a potential cause of seizures. This early attempt, however, faced strong opposition and was largely abandoned. Only in the 1930s did experimental electrophysiology begin to demonstrate that vagal stimulation could evoke responses in the sensory cortex (Bailey and Bremer, 1938). Although research on VNS remained confined to basic experimental investigations for several decades, these foundational studies played a crucial role in establishing the physiological basis for later clinical applications. Subsequent investigations showed that vagal stimulation could influence the ventral-posterior and intralaminar thalamic nuclei, suggesting that vagal afferent activity may reverberate across widespread cortical networks (Dell and Olson, 1951). Additional studies demonstrated that stimulation at frequencies of approximately 24–50 Hz induced desynchronization in the orbitofrontal cortex of cats under different experimental conditions (Bremer and Bonnet, 1951; Zanchetti et al., 1952). Complementary work further showed that direct stimulation of the solitary tract could induce EEG synchronization in cats when currents of approximately 30 Hz were applied (Magnes et al., 1961), while other studies demonstrated that stimulation frequency critically determined whether cortical effects appeared synchronizing or desynchronizing (Chase et al., 1967). Taken together, these early investigations indicate that vagal stimulation, depending on its intensity and frequency, can influence multiple brain regions and produce widespread modulation of cortical activity. These effects extend beyond simple frequency modulation and involve shifts in the balance between excitation and inhibition. A particularly influential experiment demonstrated that repetitive vagal stimulation suppressed experimental seizures in dogs (Zabara, 1992), providing a key experimental foundation for the development of the first clinical VNS protocols in human epilepsy (Terry et al., 1990).

## 3 Effects of VNS on EEG rhythms: periodic and aperiodic components in clinical studies

To synthesize the heterogeneous clinical literature on VNS-related EEG changes in drug-resistant epilepsy, the principal studies discussed in this section are summarized in Table 1.

**Table 1:** Selected clinical studies reporting VNS-related EEG changes in drug-resistant epilepsy.

Study	Population/design	EEG findings	Clinical relation
Kuba et al. (2002)	Patients with drug-resistant epilepsy. Prospective follow-up after VNS implantation.	Scalp EEG showed changes in interictal epileptiform discharges and background activity during chronic stimulation.	EEG changes were reported in parallel with clinical improvement in a subset of patients.
Marrosu et al. (2005)	Patients with refractory epilepsy undergoing long-term VNS therapy.	Quantitative EEG showed reduced theta synchronization and increased gamma power/synchronization.	EEG changes were interpreted as possible correlates of seizure reduction and improved vigilance.
Fraschini et al. (2014)	Patients with drug-resistant epilepsy undergoing VNS therapy. Observational study.	Functional connectivity analysis revealed reorganization of cortical network architecture.	Network changes were proposed as markers associated with therapeutic response.
Brázdil et al. (2019)	Pre-implant evaluation of patients considered for VNS therapy.	Baseline EEG reactivity and spectral features were analyzed as potential predictors of response.	EEG reactivity suggested possible predictive value for VNS outcome.
Coa et al. (2022)	Patients with refractory epilepsy treated with VNS.	Quantitative EEG analysis showed changes in functional connectivity and aperiodic spectral components during stimulation.	EEG patterns were discussed in relation to seizure reduction in responders.
Morrison et al. (2024)	Patients undergoing VNS with dose-dependent electrophysiological assessment.	Advanced EEG analysis identified dose-dependent modulation of oscillatory power and synchronization.	EEG signatures were proposed as candidate biomarkers related to treatment response and stimulation intensity.
Yang et al. (2024)	Prospective observational study in drug-resistant epilepsy.	Long-term changes in aperiodic EEG parameters, including spectral exponent and offset, were reported.	Some aperiodic EEG features were associated with clinical improvement.
Danthine et al. (2025)	Follow-up study in patients treated with VNS.	Quantitative EEG revealed longitudinal changes in synchronization and spectral measures.	EEG markers were explored as potential indicators of therapeutic efficacy.

Selected clinical studies reporting VNS-related EEG changes in drug-resistant epilepsy. The table summarizes representative studies addressing changes in conventional spectral measures, synchronization, connectivity, and aperiodic EEG components in relation to VNS treatment and clinical response.

### 3.1 Periodic components: EEG synchronization and desynchronization in human VNS

From the earliest clinical applications of VNS therapy, numerous studies have attempted to correlate EEG patterns with treatment outcomes, either by exploring mechanisms underlying seizure reduction (Casazza et al., 2006) or by evaluating whether EEG synchronization patterns might predict clinical response (De Vos et al., 2011). More recently, this line of research has been refined through the analysis of time-dependent changes in EEG synchronization dynamics (Danthine et al., 2025; Morrison et al., 2024). Because VNS exerts multifaceted effects on several anatomical structures involved in cortical electrogenesis, the methodological approaches used for EEG processing are critical for interpreting results. Even advanced analytical techniques cannot fully capture the entire spectrum of EEG changes produced by VNS. Another important factor concerns the temporal window selected for analysis. In most studies, VNS outcomes are evaluated one or more years after device implantation, making longitudinal consistency in EEG measurements particularly relevant. Equally important is the choice of mathematical approaches used to characterize relationships between time- and frequency-domain EEG signals. Although a detailed discussion of advanced EEG analytical methods lies beyond the scope of this review, one illustrative example involves the use of nonlinear synchronization measures (Quiroga et al., 2002). Unlike traditional coherence, cross-correlation, or mutual information approaches, nonlinear synchronization analyses may better capture complex interdependencies between EEG signals. Using such methods, chronic VNS has been reported to significantly suppress lower-frequency EEG synchrony while enhancing higher-frequency activity. In particular, synchronization within the theta band (4–8 Hz) decreases following long-term VNS treatment (Marrosu et al., 2005). This finding is notable because excessive theta synchronization is frequently associated with drowsiness and reduced vigilance, conditions that may also be exacerbated by anti-seizure medications. In contrast, gamma-band activity (20–50 Hz) shows a marked increase with VNS therapy, with significant increases observed in both gamma power and synchronization. Interestingly, intermediate frequencies such as the alpha band (8–13 Hz) do not appear to change significantly, suggesting that the electrophysiological effects of VNS may be most pronounced at the lower (theta) and higher (gamma) ends of the EEG spectrum. Similarly, no consistent alterations in delta background rhythms have been reported, apart from a general desynchronizing trend reflected by reduced pathological delta synchronization over time. These findings are broadly consistent with earlier animal studies. Classic experiments in cats demonstrated that vagal afferent stimulation could desynchronize cortical EEG activity, producing faster and more alert cortical patterns under specific stimulation conditions. Taken together, these results suggest that long-term VNS may shift epileptic brain activity away from slower, highly synchronized theta rhythms toward higher-frequency, more desynchronized states characterized by enhanced gamma oscillations. Because gamma oscillations are often associated with attentive and cognitively engaged brain states, these findings have raised the possibility that VNS may not only reduce seizure frequency but also contribute to improvements in mood (Elger et al., 2000), alertness, and quality of life (Dodrill and Morris, 2001; Galli et al., 2003).

### 3.2 Aperiodic component analysis and spectral exponent dynamics

More recently, comprehensive analyses of EEG changes following VNS have incorporated information derived from aperiodic dynamics. Traditional power spectral analysis assumes that oscillatory peaks rest on a flat baseline; however, neural spectra follow an approximate 1/f distribution. The spectral exponent (slope) of this distribution becomes steeper with increased inhibition or reduced neural firing, while the offset reflects broadband power. If not properly disentangled, oscillatory peaks from this aperiodic background may lead to misinterpretation. For example, an apparent increase in gamma power may partly reflect a flatter (shallower) exponent rather than a true oscillatory gain (Gerster et al., 2022). One study comparing pre-implant with one-year post-implant EEG recordings reported significant reductions in both exponent and offset across frontal, temporal, and occipital regions in responders, whereas non-responders showed increases in these measures (Coa et al., 2022). In this study changes in aperiodic metrics correlated with clinical outcomes, with exponent reduction (flatter slope) in responders interpreted as a shift toward reduced cortical inhibition and lower neural noise, potentially reflecting network renormalization. These findings are consistent with earlier results obtained using conventional frequency-based EEG analysis (Fraschini et al., 2014). A larger study (Yang et al., 2024) reported that, among responders, both exponent and offset decreased only at long-term follow-up (>3 years). Although effect sizes were moderate, greater reductions in the exponent predicted stronger seizure reduction, while non-responders showed no consistent changes. Importantly, age did not significantly influence aperiodic shifts, suggesting that VNS-driven plasticity rather than aging accounted for the observed effects. Converging evidence also comes from intracranial responsive neurostimulation (RNS) in mesial temporal lobe epilepsy (Charlebois et al., 2024). Greater circadian modulation of the aperiodic exponent during the first 3 months of therapy appears to predict improved seizure reduction at 1 year. Although stimulation modalities differ, these findings support the idea that aperiodic dynamics capture clinically relevant shifts in network excitability across neuromodulation approaches. Nevertheless, caution is warranted in interpreting these results, as several methodological issues remain. First, parameterization is highly sensitive to the chosen fitting range, particularly when low-frequency delta activity is prominent. Fitting bounds must therefore be selected carefully to avoid bias in slope estimation (Hosseini et al., 2021). Second, muscle and stimulation artifacts can significantly affect high-frequency components: EMG contamination may mimic exponent flattening, making rigorous artifact rejection and monitoring essential. In addition, VNS stimulation at approximately 30 Hz produces harmonics that should be removed, for example through notch filtering, prior to spectral fitting to prevent biased offset estimates. Longitudinal consistency is also critical, as changes in the exponent often emerge over extended periods. Consistent amplifier settings and referencing schemes across sessions are therefore required. Taken together, these technical considerations highlight that aperiodic component analysis remains complex and context-dependent (Donoghue, 2025). Future research should aim to clarify causal mechanisms, refine spectral fitting methodologies, and integrate aperiodic features into multivariate models suitable for real-time clinical decision-making. Such developments will be essential for translating spectral findings into next-generation VNS systems capable of adaptively stabilizing cortical excitability. At the same time, exclusive reliance on aperiodic analysis risks oversimplification. A more robust approach is to integrate periodic and aperiodic measures, interpreting VNS-induced desynchronization as a broadband shift in brain dynamics. Future studies should therefore routinely report both raw and aperiodic-adjusted band power to provide a more accurate characterization of EEG changes under VNS. Finally, combining these analyses with neurochemical data may further improve interpretation and support optimization of ASM treatment.

## 4 Correlation of neurochemical variants induced by VNS and EEG activity

Because the proposed relationship between VNS and EEG changes likely depends on multiple interacting transmitter systems rather than a single pathway, the principal neurochemical systems discussed here are summarized in Table 2.

**Table 2:** Principal neurochemical systems implicated in VNS effects and their putative relevance to EEG modulation.

System	Anatomical relay	Evidence	EEG relevance
Noradrenergic	Locus coeruleus projections activated via NTS pathways.	Animal and human studies show VNS-induced activation of LC neurons and increased norepinephrine release.	May modulate cortical excitability and synchronization, with possible influence on oscillatory activity, particularly theta rhythms and arousal-related dynamics.
Serotonergic	Raphe nuclei receiving input from NTS circuits.	Experimental studies support increased serotonergic transmission during VNS.	May influence cortical rhythms, mood-related EEG changes, and large-scale network stability.
Cholinergic	Basal forebrain and brainstem cholinergic pathways.	Neuromodulation studies suggest interaction between vagal input and cholinergic systems.	May contribute to cortical arousal, desynchronization, and modulation of theta- and alpha-related activity.
GABAergic	Local inhibitory interneuron networks within cortical and limbic circuits.	VNS may enhance inhibitory tone through direct and indirect neuromodulatory pathways.	May contribute to stabilization of cortical activity and reduction of epileptiform discharges.
Neurotrophic pathways (BDNF)	Neuroplasticity mechanisms activated by neuromodulatory signaling.	Experimental studies show VNS-induced changes in BDNF expression and synaptic plasticity.	May support long-term network remodeling and indirectly influence EEG connectivity, synchronization, and plasticity-related electrophysiological changes.

The table summarizes the main transmitter and neurotrophic systems discussed in the literature, the principal anatomical relays involved, the nature of the supporting evidence, and their possible electrophysiological and clinical implications.

### 4.1 Linking EEG modulation with neurotransmitter systems

The extensive literature on EEG following VNS suggests that significant modifications in electrophysiological patterns, whether reflected in rhythmic activity, aperiodic components, or both, are associated with the effects of vagal stimulation on distributed brain networks mediated by multiple neurotransmitters, neuromodulators, and neurotrophic factors. The interaction between stimulation parameters (such as current intensity and pulse duration), their effects on neurochemical systems, and the corresponding EEG changes represents a complex and only partially understood field of investigation. EEG remains a relatively inexpensive yet highly informative tool for assessing VNS effects, particularly when interpreted within a broader neurochemical framework.

### 4.2 The nucleus of the solitary tract and its broad network influence

Converging evidence indicates that the effects of VNS are primarily mediated by the nucleus of the solitary tract (NTS), whose role extends well beyond its classical involvement in visceral regulation (Huang et al., 2025). The NTS also participates in nociceptive modulation through its projections to the periaqueductal gray, cerebellum, hypothalamic and thalamic nuclei, the central nucleus of the amygdala, and the limbic reticular formation (Patros et al., 2025). These widespread connections help explain how VNS exerts broad modulatory effects on EEG dynamics and cortical excitability. Neurochemical studies further indicate subtle yet significant changes across multiple neurotransmitter systems that directly and indirectly influence brain rhythms. Given the extensive projections of the NTS, mapping all possible correlations between neurochemical modulation and EEG variation remains challenging. Nevertheless, certain systems appear particularly relevant for understanding the balance between inhibition and excitation reflected in EEG activity. Of particular importance are NTS projections to the locus coeruleus (LC) and the raphe nuclei (RN), the primary sources of noradrenergic and serotonergic innervation in the brain.

### 4.3 Role of noradrenergic and serotonergic pathways in VNS

Seminal studies have demonstrated that NTS projections to the LC enhance both noradrenergic and serotonergic transmission (Dorr and Debonnel, 2006) and are critical for suppressing epileptic spike-and-wave activity, as VNS loses its antiseizure effects following lesions of this pathway (Krahl et al., 1998). These findings suggest that noradrenaline released by the LC is a key mediator of VNS effects, even during long-term stimulation, contributing to reduced synchronization of epileptogenic activity and shaping EEG patterns. Experimental evidence further supports this role: depletion of noradrenaline through 6-hydroxydopamine lesions reverses these effects (Kostrzewa and Jacobowitz, 1974). Noradrenaline may exert anti-kindling effects through post-synaptic alpha-2 receptors, delaying seizure progression (Gellman et al., 1987). It is therefore plausible that VNS modulates the noradrenergic system to inhibit or slow the spread of after-discharges, a key process in both experimental and clinical epileptogenesis. In addition, the NTS projects directly to the dorsal raphe nucleus (Patros et al., 2025), the brain’s principal serotonergic center, which strongly influences hippocampal activity. Administration of the serotonergic agonist 5-OH-DPAT induces rhythmic theta activity in animal models (Marrosu et al., 1996), supporting a role for serotonin in this oscillatory pattern. Theta activity is closely linked to long-term potentiation (LTP), and VNS has been shown to enhance LTP in freely moving rats (Zuo et al., 2007). These effects may act synergistically with noradrenergic modulation. Moderate VNS stimulation increases discharge rates in LC neurons (Groves et al., 2005) and enhances activity in both hippocampal and cortical regions (Roosevelt et al., 2006). Recent findings also suggest that LC-mediated pupil dilation in response to VNS may serve as a biomarker of neuromodulatory engagement (Sánchez et al., 2025). In parallel, microdialysis studies have demonstrated increased noradrenaline concentrations in fronto-parietal regions of freely moving rats following VNS (Follesa et al., 2007), supporting previously reported effects on mood, alertness, and quality of life.

### 4.4 Hippocampal synchrony, periodic EEG modulation, and temporal lobe epilepsy

The hippocampus, characterized by highly synchronous pyramidal networks, plays a central role in mesial temporal lobe epilepsy (TLE), the most common form of focal epilepsy, partly due to its selective vulnerability and neuronal loss (Gloor, 1990). VNS appears particularly effective in reducing epileptiform discharges in patients with TLE (Terry et al., 1990). Several experimental findings suggest that VNS may counteract key pathophysiological mechanisms underlying this condition. For example, hippocampal noradrenaline levels have been shown to correlate with VNS efficacy (Raedt et al., 2011), and chronic VNS has been associated with increased hippocampal neurogenesis (Biggio et al., 2009). Another important mechanism involves modulation of theta-band synchrony via muscarinic receptors (Nichols et al., 2011), supporting a role for acetylcholine (ACh) in VNS effects. ACh modulates hippocampal theta activity by influencing specific neuronal subtypes. In particular, “theta-on” cells may shift from tonic to phasic firing, a transition critical for rhythm generation and for linking intracellular membrane properties with extracellular EEG signals (Bland and Colom, 1988). Experimental studies suggest that VNS can promote this shift, thereby reorganizing network synchronization from pathological toward more physiological patterns (Arnolds et al., 1980; Zaveri et al., 2001). This mechanism is especially relevant in the CA3 region, which is highly involved in seizure generation and propagation (Song et al., 2018). More broadly, modulation of hippocampal synchrony through serotonergic and cholinergic pathways may influence large-scale brain networks. Because hippocampal oscillations are broadcast to circuits involved in memory, spatial orientation, and cognitive integration, VNS-induced changes may extend to multiple cortical and subcortical regions (Buzsáki, 2002).

### 4.5 Aperiodic EEG shifts and neurochemical correlates

Although systematic investigation remains limited, available evidence suggests that serotonergic, noradrenergic, and cholinergic modulation under VNS may contribute to changes in aperiodic EEG components. Experimental studies have shown correlations between the 1/f exponent and noradrenaline levels, as well as with pupil diameter, supporting a link between arousal-related neuromodulation and aperiodic EEG structure (Pertermann et al., 2019). By contrast, the roles of acetylcholine and serotonin in shaping the aperiodic component remain less clearly defined. While direct studies are lacking, ACh is known to promote alpha rhythm generation and cortical desynchronization, suggesting that it may flatten the aperiodic exponent by increasing higher-frequency activity. A similar hypothesis may apply to serotonin, which could potentially steepen the exponent by enhancing lower-frequency activity. At present, however, these interpretations remain speculative and require direct experimental validation.

### 4.6 GABA, BDNF, and neurotrophic contributions

The gamma-aminobutyric acid (GABA) system represents one of the most critical pathways involved in VNS effects, given its central role in inhibitory signaling and epilepsy pathophysiology (Treiman et al., 2016). Successful VNS, independently of ASM adjustments, has been shown to modify cortical GABA-A receptor expression in both humans (Marrosu et al., 2003) and experimental models (Neese et al., 2006). These effects may be partially mediated by brain-derived neurotrophic factor (BDNF). Experimental studies support this relationship. In models where epileptic GABA receptors were expressed in frog oocytes, repetitive GABA application led to receptor rundown, whereas exposure to BDNF increased current amplitude and reduced this effect (Palma et al., 2005). BDNF has also been shown to reduce cellular damage in hippocampal lesions induced by prolonged seizures (Paradiso et al., 2009) and to promote neurogenesis, including the generation of GABA-somatostatin interneurons, which are essential for dendritic inhibition and whose loss contributes to epileptogenesis (Binaschi et al., 2003). VNS has been shown to increase BDNF levels across several brain regions (Follesa et al., 2007). Although direct links between BDNF and EEG remain incompletely defined, its role in inhibitory signaling suggests downstream effects on network organization and electrogenesis. VNS also influences sleep–wake regulation, likely through GABAergic mechanisms. Clinically, it reduces daytime sleepiness and modifies sleep EEG architecture (Malow et al., 2001). Experimental studies in cats have shown increased slow-wave sleep and spindle activity. Because reduced spindle activity is associated with increased seizure susceptibility (Roebber et al., 2022), these findings may have clinical relevance. Taken together, these observations highlight the interconnected effects of VNS on neurotransmitters, neuromodulators, neurotrophic processes, and EEG activity. Importantly, GABAergic effects do not always align straightforwardly with aperiodic EEG measures if exponent steepening is interpreted simply as increased inhibition. Recent pharmacological evidence supports a more nuanced interpretation. For example, selective GABA-A antagonism (e.g., picrotoxin or bicuculline) paradoxically increases the spectral exponent in awake rodents, suggesting that behavioral state and neural noise also influence this metric (Gerster et al., 2022). For this reason, interpretation of VNS-related aperiodic EEG changes requires a multimodal framework that integrates electrophysiological and neurochemical data. Overall, these findings support the view that VNS engages multiple neuromodulatory systems capable of shaping large-scale cortical dynamics. These effects may be reflected in measurable EEG signatures and may provide a mechanistic bridge between neurochemical modulation and clinical outcomes. A conceptual summary of these relationships is illustrated in the accompanying flow chart.

## 5 Future directions: AI-driven EEG analysis and adaptive VNS

To translate these mechanistic and electrophysiological observations into clinically useful tools, possible computational approaches and their current limitations are summarized in Table 3.

**Table 3:** Computational approaches potentially relevant for VNS-related EEG biomarker development.

Approach	EEG Features / Inputs	Potential Application	Current Evidence Level	Main Limitations
Supervised EEG classification	Spectral power, oscillatory rhythms, connectivity metrics	Identification of electrophysiological patterns associated with VNS response	Moderate (mainly small cohort studies)	Limited generalizability, variability across datasets
Connectivity-based prediction	Functional connectivity, network topology measures	Prediction of treatment response based on baseline or longitudinal network organization	Emerging	Lack of standardization, sensitivity to preprocessing choices
Aperiodic feature analysis	Spectral exponent and offset	Characterization of excitation–inhibition dynamics and their modulation by VNS	Emerging	Interpretation still debated, limited clinical validation
Seizure-state detection	Time-resolved EEG features (pre-ictal/ictal markers)	Real-time monitoring of seizure-related activity during VNS therapy	Established in epilepsy, limited in VNS-specific context	False positives, patient-specific variability
Closed-loop adaptive stimulation	Continuous EEG monitoring, algorithm-based decision systems	Development of responsive VNS systems adapting stimulation parameters	Experimental / early-stage	Technical complexity, regulatory challenges, need for robust biomarkers

The table outlines candidate analytical strategies for prediction, monitoring, and adaptive stimulation, together with likely input features, current evidence level, and major limitations.

### 5.1 General perspective

Neurophysiology and neurochemistry are among the fields in which AI-based computational approaches have shown particularly strong potential (Easttom, 2024). By leveraging these tools (LeCun et al., 2015), AI may contribute not only to a deeper understanding of epilepsy more broadly (An et al., 2020), but also to a more refined interpretation of the mechanisms and therapeutic effects of VNS. Within this framework, several future directions can be envisioned for the application of AI to VNS-related EEG biomarker development.

### 5.2 Automated EEG classification and machine learning

EEG analysis has greatly benefited from the introduction of machine learning techniques, with different models offering distinct advantages depending on the application and dataset characteristics. It is therefore essential to identify the most appropriate computational strategy for each specific objective. One promising direction involves the automated classification of EEG patterns using deep neural networks and related approaches for EEG signal decomposition (Lopes et al., 2022), applied to VNS-related neurophysiological changes. Because VNS induces distinct modifications in spectral content and connectivity, machine learning systems could potentially be trained to recognize and classify these responses. Candidate features may include changes in band power across the delta, theta, alpha, beta, and gamma ranges (Fraschini et al., 2013), as well as inter-regional coherence (Wijayanto et al., 2022) and phase-synchrony measures such as the phase-lag index (PLI) for quantifying functional connectivity (Stam et al., 2007). Supervised classifiers, including support vector machines and neural networks, could be trained on labeled clinical datasets to predict outcomes such as responder versus non-responder status. Clinically, such tools could support real-time monitoring of post-stimulation EEG to determine whether the brain is responding as expected, as has already been explored in other brain stimulation modalities (Shirinpour et al., 2025). This approach may allow earlier detection of likely non-response, well before long-term clinical outcomes become apparent, and may also help identify electrophysiological response subtypes requiring personalized stimulation strategies.

### 5.3 Predictive modeling of VNS responders

Another major application of AI is the prediction of which patients are most likely to respond to VNS based on pre-implant or early post-implant EEG features. Several EEG measures, including frequency-band ratios, hemispheric symmetry, connectivity patterns, and complexity metrics, have historically been associated with treatment outcomes. For example, patients showing increased gamma-band activity or more asymmetric and desynchronized interictal EEG patterns have been reported to be more likely to respond to VNS (Zabara, 1992; Terry et al., 1990; Brázdil et al., 2019). AI models, particularly deep learning systems trained on sufficiently large datasets, could potentially identify more complex EEG patterns associated with treatment success (Dissanayake et al., 2021). Predictive scores have already been developed in other epilepsy-related clinical settings, such as presurgical evaluation, using clustering approaches including K-means methods (Babtain et al., 2024). A comparable strategy could be applied to VNS in order to reduce unnecessary implantation in likely non-responders and prioritize candidates with more favorable EEG markers. In addition, predictive modeling could be extended to early post-implant EEG, enabling more timely optimization of stimulation parameters based on short-term biomarkers, such as high-frequency oscillations or EEG microstates reflecting large-scale network dynamics (Huang et al., 2025), rather than waiting for delayed clinical outcomes.

### 5.4 Closed-loop adaptive VNS systems guided by EEG

Perhaps the most transformative future direction lies in the development of closed-loop VNS systems capable of dynamically adjusting stimulation parameters in real time while minimizing side effects. Adaptive closed-loop VNS has already been explored not only in epilepsy, but also in other conditions such as cardiovascular disease (Ottaviani et al., 2022) and spinal cord injury (Kilgard et al., 2025). Current clinical VNS devices, however, operate mainly in open-loop mode, delivering fixed intermittent pulses or, in some cases, responding to indirect physiological signals such as heart rate. By contrast, an AI-enhanced closed-loop system would continuously monitor EEG activity and modify stimulation parameters according to the current electrophysiological state of the brain (Mathews et al., 2025). For example, if EEG recordings showed pathological synchronization, such as bursts of theta activity or spike–wave discharges, the device could automatically increase stimulation intensity or deliver additional pulses to disrupt the emerging abnormal state. Conversely, if the EEG indicated a relatively healthy desynchronized state with preserved fast-frequency activity, stimulation could be reduced, thereby conserving battery life and limiting unnecessary stimulation. In addition, deep learning algorithms could be trained to detect pre-seizure states or early hypersynchrony, while reinforcement learning approaches could optimize stimulation strategies over time by learning how specific changes in VNS parameters influence EEG patterns, with the goal of maximizing the duration during which EEG biomarkers remain within a desired therapeutic range (Kostov et al., 2025). Over time, such a closed-loop system could learn the individual EEG profile of each patient and adapt stimulation timing and intensity accordingly, bringing VNS closer to a truly personalized neuromodulation strategy.

## 6 Challenges, perspectives, and conclusion

Over the past three decades, research has substantially advanced our understanding of how VNS modulates brain activity in epilepsy, primarily through measurable EEG changes and, more recently, through reorganization of functional connectivity associated with reduced seizure frequency and improved quality of life. These findings underscore the importance of investigating the neurochemical mechanisms underlying EEG signatures, not only to better understand the mechanisms of VNS itself, but also, through deeper insight into neurotransmitter and neuromodulator dynamics, to inform more rational antiseizure medication strategies. Despite these advances, important limitations remain in the current evidence base. Direct studies simultaneously measuring EEG dynamics and neurochemical changes during VNS in humans remain scarce, and much of the mechanistic understanding still relies on animal models or indirect physiological markers. In addition, methodological heterogeneity in EEG acquisition and analysis, including differences in spectral parameterization and connectivity metrics, complicates cross-study comparisons and limits the identification of robust biomarkers predictive of treatment response. Looking ahead, the integration of machine learning and AI into VNS research holds considerable promise for advancing personalized neuromodulation. Automated EEG-based classifiers and predictive models may improve patient selection and treatment optimization, while intelligent closed-loop stimulation systems may enhance efficacy by dynamically counteracting epileptic activity. Such developments, when integrated with advances in neurochemical research, align with the broader framework of precision medicine and have been identified as important next steps in neuromodulation research (Austelle et al., 2024). Early progress is already visible. For example, implantable systems have been developed that trigger VNS when ictal tachycardia is detected as a proxy for seizure onset. Incorporating EEG monitoring into such systems would represent a major advance by directly assessing brain activity rather than relying solely on peripheral physiological markers (Fischer et al., 2015). Taken together, these developments suggest that the convergence of EEG-based insights and AI-driven tools could transform VNS from a largely empirical therapy into a more responsive and data-informed intervention. A closed-loop EEG-guided VNS system could aim not only to interrupt seizures, but also to continuously optimize brain network function, for example by sustaining reductions in theta-band coherence or maintaining gamma activity above a therapeutic threshold, both of which have been associated with seizure control and alertness. An additional, more speculative direction emerges from experimental studies on cognitive enhancement (Olsen et al., 2022). If coupled with more refined neurochemical monitoring, such approaches might also help counteract some of the cognitive side effects associated with antiseizure medications. One could envision a closed-loop VNS system capable of delivering stimulation bursts when EEG markers of attentional decline, such as reductions in mid-frontal theta or gamma activity, are detected, thereby supporting focus during cognitively demanding tasks through the same neuromodulatory shifts that underlie improved vigilance. Important challenges remain, including safety, algorithm validation, clinical generalizability, and regulatory approval. Nevertheless, the integration of real-time EEG analysis with adaptive neurochemical insight represents a promising frontier at the intersection of AI, engineering, and neuroscience. Ultimately, this approach could transform VNS into a smarter neuromodulatory prosthesis, capable of stimulating only when needed to help maintain more favorable brain states. Collectively, these advances may not only deepen our understanding of VNS mechanisms, but also promote closer integration between research and clinical practice, thereby broadening the therapeutic potential of this technology.
